# Analysis of AI-Generated Patient Education Guides for Urological Conditions: A Comparative Study Between ChatGPT and Gemini

**DOI:** 10.7759/cureus.99969

**Published:** 2025-12-23

**Authors:** Santhosh Kumar Mohan Kumar, Niranjana Ananthan

**Affiliations:** 1 General Surgery, Southampton General Hospital NHS Foundation Trust, Southampton, GBR; 2 General Medicine, Southampton General Hospital NHS Foundation Trust, Southampton, GBR

**Keywords:** artificial intelligence, chatgpt, gemini, patient education, urology

## Abstract

Introduction

Artificial intelligence (AI) chatbots are increasingly being used to create patient education guides (PEGs). However, there are gaps in the literature comparing the latest version in terms of readability, reliability, and similarity. The aim of this study was to compare PEGs generated by ChatGPT 5.1 (OpenAI, San Francisco, California, US) and Gemini 3 Pro (Google LLC, Mountain View, CA, USA) for five common urological conditions, kidney stone, urinary tract infection, urinary retention, erectile dysfunction, and benign prostatic hyperplasia, across these domains.

Methods

This cross-sectional study analysed PEGs generated by both AI chatbots for five common urological conditions using identical prompts. Readability was assessed using the Flesch Reading Ease Score and Flesch-Kincaid Grade Level. Reliability and similarity were assessed using a modified DISCERN score and Turnitin, respectively. Statistical comparison was performed using the Mann-Whitney U test.

Results

None of the evaluated characteristics showed a statistically significant difference between the PEGs generated by AI chatbots.

Conclusion

PEGs generated by both AI chatbots exceeded the recommended reading level, demonstrated limited originality, and showed moderate reliability, highlighting the need for professional oversight. Continued refinement of AI chatbots is necessary before integrating AI-generated PEGs into routine patient education.

## Introduction

Five common urological conditions were selected for the study from the 'frequent topics' list of the National Institute of Diabetes and Digestive and Kidney Diseases [1]. Conditions are kidney stones, urinary tract infection (UTI), urinary retention, erectile dysfunction (ED), and benign prostatic hyperplasia (BPH).

Kidney stones are solid deposits made of minerals and salts that form inside the kidney. UTI is an infection affecting any part of the urinary system. Urinary retention is the inability to fully empty the bladder. ED is the inability to achieve and/or maintain an erection. BPH is a non-cancerous growth of the prostate gland [2-6].

Patient education is an important part of the treatment of urological diseases. Patient education will lead to early recognition of symptoms, improved adherence to treatment, fewer complications, and consequently, it will lead to better clinical outcomes [7]. Nowadays, artificial intelligence (AI) is being used more frequently in patient education to provide personalised information. The ability of AI to customise educational content based on a patient’s data, engage patients in real-time, and generate responses that are appropriate for different literacy levels or preferred languages is the sole reason. This personalised approach improves understanding of condition and treatment options, encourages them to make informed decisions, and improves compliance [8].

We selected two AI chatbots, Gemini 3 Pro (Google LLC, Mountain View, CA, USA) and ChatGPT 5.1 (OpenAI, San Francisco, California, US), due to their popularity and mentionable performance. ChatGPT and Gemini are based on different architectures and trained using different methods. Gemini supports multimodal input, allowing it to process and generate images or other media in addition to text, making it suitable for tasks with varied data, such as patient education that includes visuals, while ChatGPT excels at generating high-quality textual interactions, offering fluent conversational responses and strong performance in writing and coding tasks [9].

Prior studies have evaluated previous versions of ChatGPT and Gemini for the generation of patient education guides (PEGs) [10,11]. However, to our knowledge, there is a lack of studies on the performance of newer versions of ChatGPT and Gemini.

The aim of this study was to conduct a metric-based comparison of the effectiveness of PEGs generated by ChatGPT 5.1 and Gemini 3 Pro across three key parameters: readability, assessed using the Flesch Reading Ease Score (FRES) and the Flesch-Kincaid Grade Level (FKGL); reliability, evaluated using a modified DISCERN score; and similarity, measured using Turnitin’s similarity index for five urological conditions: kidney stones, UTI, urinary retention, ED, and BPH.

## Materials and methods

Study design

This cross-sectional study was conducted to evaluate the readability, reliability, and similarity of AI-derived PEGs for common urological conditions. As neither human nor animal subjects were involved, and all data were exclusively derived from two AI models, ChatGPT 5.1 and Gemini 3 Pro, the study did not require ethical approval. The received responses were analysed digitally using appropriate methods and software.

Data collection

We conducted the study over one week (November 23-30, 2025), where two AI chatbots were issued prompts: ChatGPT 5.1 [12] and Gemini 3 Pro [13]. A newly created account was used for each AI chatbot with default settings to obtain standard responses and exclude any bias from previous interactions. A new 'chat' was started for each condition, with both AI chatbots given the same prompts: "Write a patient education guide for [name of condition]." All prompts were given to both AI chatbots on a single day (November 25, 2025). The AI chatbot's responses for each prompt were pasted into a separate Microsoft Word (Microsoft Corp., Redmond, WA, USA) document for analysis.

Assessment tools

Three standardised tools were employed to assess the generated content.

Readability Assessment

The readability of the AI-generated content was assessed using the FRES [14] and the FKGL [15]. Readability was measured using the FRES value (on a scale from 0 to 100, with higher values indicating easier readability). The FKGL estimates the years of education necessary to comprehend the text. These metrics are frequently used to evaluate medical literature and aim to make it accessible to patients with various levels of literacy [16]. The AI responses were pasted into WebFX (a free online tool) for automatic readability scoring [17].

Reliability Assessment

The reliability of the generated AI content was assessed based on a modified form of the DISCERN score, which is used to assess the reliability of health information, as performed in prior studies [10]. The modified DISCERN score has five scoring criteria, each involving binary scoring (0 or 1), with cumulative scores ranging from a minimum of 0 to a maximum of 5. Higher scores reflect better reliability. The responses were scored by two observers who independently assessed the images. Any discrepancies were resolved through a consensus decision before the final analysis.

Similarity Assessment

To assess the similarity of AI-generated content, Turnitin's text-matching software was used [18]. A Word document created for each condition was uploaded to Turnitin, and a similarity report was generated, resulting in an overall similarity index (OSI), which reflects the amount of text copied from sources. The default settings of Turnitin were used for this analysis. This helped to determine whether the AI chatbots were creating new content or regurgitating existing publications.

Statistical analysis

The readability scores (FRES and FKGL), reliability scores (modified DISCERN), and similarity percentages (Turnitin OSI) were analysed using SPSS version 24 (IBM Corp., Armonk, NY). The medians of the readability scores, reliability scores, and similarity percentages between ChatGPT and Gemini were compared using the Mann-Whitney U test. A p-value of <0.05 was considered statistically significant.

## Results

The properties of PEGs generated by ChatGPT and Gemini are shown in Table 1.

**Table 1:** Characteristics of the patient education guides generated by ChatGPT and Gemini IQR, interquartile range.

Parameters	Median (IQR)		U statistic	p-value
	ChatGPT	Gemini		
Grade	12.9 (12.6-13.3)	10.3 (8.7-10.4)	3	0.056
Ease	33.1 (31.2-35.5)	44.6 (41.9-50.9)	4	0.095
Reliability	3.0 (3.0-3.0)	3.0 (3.0-3.0)	10	0.69
Similarity	56.0 (54.0-58.0)	42.0 (37.0-62.0)	11	0.841

**Figure 1:**
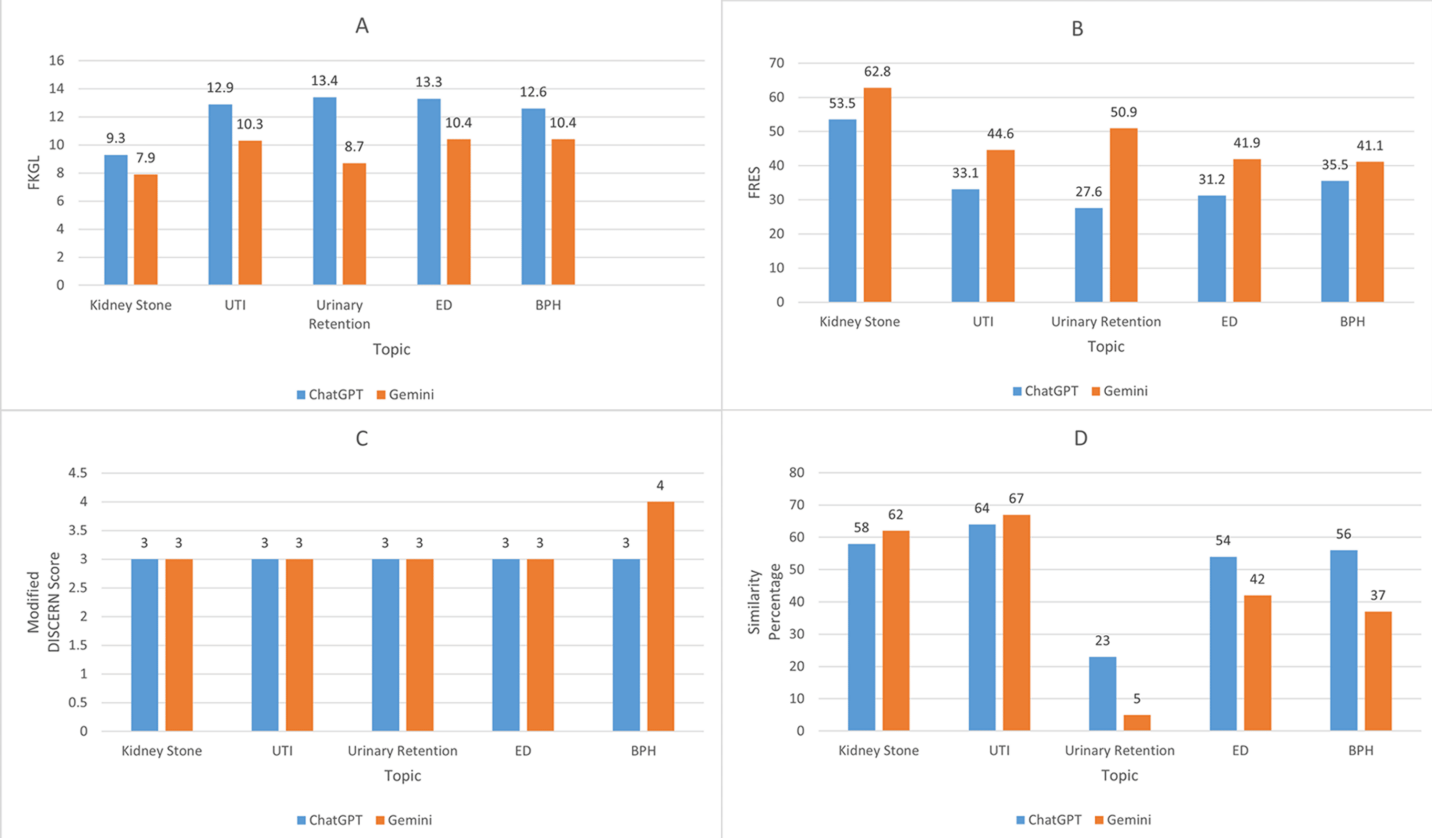
Comparison of FKGL (A), FRES (B), modified DISCERN score (C), and similarity percentage (D) for patient education guides generated by ChatGPT and Gemini FKGL, Flesch-Kincaid Grade Level; FRES, Flesch Reading Ease Score.

Bar graph comparison of the FKGL, FRES, modified DISCERN score, and similarity percentage for ChatGPT and Gemini-generated PEGs is presented in Figure 1.

PEGs generated by Gemini on kidney stones had the lowest grade level, while the PEG generated by ChatGPT on urinary retention had the highest grade level (Figure 1A). Correspondingly, out of the five urological conditions, Gemini had the highest ease score for the PEG on kidney stones, whereas the ChatGPT-generated PEG on urinary retention had the lowest ease score (Figure 1B). The PEGs generated by Gemini on BPH had the highest modified DISCERN score, while the rest of the PEGs produced by both AI chatbots had the same score (Figure 1C). The Gemini-generated PEG on urinary retention had the lowest similarity percentage, while the PEG generated on UTI by Gemini had the highest (Figure 1D).

## Discussion

Our study comparing ChatGPT 5.1 and Gemini 3 Pro generated PEGs on kidney stones, UTI, urinary retention, ED, and BPH found no statistically significant differences in all parameters.

AI-generated customised content adds value to patient education by providing in-depth insights into medical topics. These AI applications, including chatbots, can be used in teaching and learning contexts to make the content more interesting and accessible by simplifying complex health subjects or responding to patient questions in real-time. AI has made it easier to digest medical information from the internet, which is not easily readable or comprehensible [19]. Our study evaluated the readability of guides generated by Gemini and ChatGPT. The complexity of the information was measured using the FKGL. The recommended grade level is six or lower [20]. The median grade levels for ChatGPT and Gemini are higher than the recommended standard. This finding is consistent with a previous study by Saji et al., which found that no AI chatbot-generated PEG is written at or near the recommended grade level for patients [21].

The median modified DISCERN score was the same for both Gemini and ChatGPT and was of moderate quality with room for improvement. This result is similar to the study of Thayappa et al., which reported moderate reliability of PEGs produced by Gemini and ChatGPT [22]. This is because of the absence of references in the PEGs generated by the AI chatbots.

The AI chatbots could replicate the same data that is used to train the model. When AI-generated responses are replicas of existing literature without proper citation or acknowledgment, they undermine scientific integrity. Our study revealed that the median similarity percentage was slightly higher for ChatGPT in comparison to Gemini, even though both chatbots have a median similarity score above 40%. A study by Reshi et al. had similar findings, where ChatGPT showed higher similarity than Gemini in creating patient information leaflets [23]. Median similarity of PEGs generated by both AI chatbots exceeded the recommended similarity percentage, which was less than 20%, even though Gemini’s PEGs on urinary retention have only 5% similarity [24].

AI chatbots do not actually verify the scientific integrity of the data that is used to train them, which can affect the quality of the end result. Additionally, the chatbots are not updated frequently and may produce responses from outdated information. Another limitation is that articles requiring payment or subscription cannot be accessed, so the AI tool can only extract knowledge from open literature or free full-text articles, meaning it may not always provide the latest medical information [25].

Limitation

Our study only evaluated two versions of AI chatbots and five urological conditions. Furthermore, the research did not evaluate other patient-specific factors, such as comorbidities and medical histories, which may reveal how well AI-generated PEGs can adapt to patient-specific conditions. Evaluation of PEGs by patients was not incorporated. The scope of our study was limited to English language materials, which limits the global implications of our findings. Furthermore, this study lacked a comprehensive fact-checking process, and the generated guides were not verified for accuracy. Our study only assessed AI-generated PEGs on a single day, with similar prompts, but the stochastic nature of AI responses, along with the lack of standardised prompts and model configurations, may have caused variations and inconsistency in output quality. The modified DISCERN scores' inter-rater reliability was also not explicitly discussed, and there was no clear guidance on how to handle ambiguous or borderline answers, adding subjectivity to reliability assessment.

## Conclusions

This study compared PEGs generated by two AI chatbots on common urological conditions in terms of readability, reliability, and similarity. The results revealed that there was no significant difference in readability, reliability, and similarity percentage between the two AI chatbots that generated PEGs. However, it is important to note that this analysis covered only two versions of AI chatbots, ChatGPT 5.1 and Gemini 3 pro, had a small sample size, and was exploratory in nature. Therefore, the results of the study may not generalise to all AI chatbots or versions. Future studies should include additional AI chatbots and versions to achieve better results and broader generalisation of the research.

Although PEGs generated using AI chatbots are considerable, they rarely meet the high standards required for patient education due to low ease of reading, lack of references, and lack of originality. This can result in information that is potentially confusing or misleading to patients. AI-generated PEGs should be supervised by a professional to make sure they are correct and up to date with current medical information. Future studies should focus on improving the AI chatbots and developing effective evaluation strategies to incorporate them into the medical field.
